# Supplementary material for: Unpredictable Repeated Stress in Rainbow Trout (Oncorhynchus mykiss) Shifted the Immune Response against a Fish Parasite
Source: Biology (Basel). 2024 Sep 27;13(10):769. doi: 10.3390/biology13100769 (PMC11504028; doi:10.3390/biology13100769)
Supplement: Supplementary file 1 [file biology-13-00769-s001.zip › biology-3198885-supplementary.pdf]

Supplementary Materials

Ich eDNA level and estimate

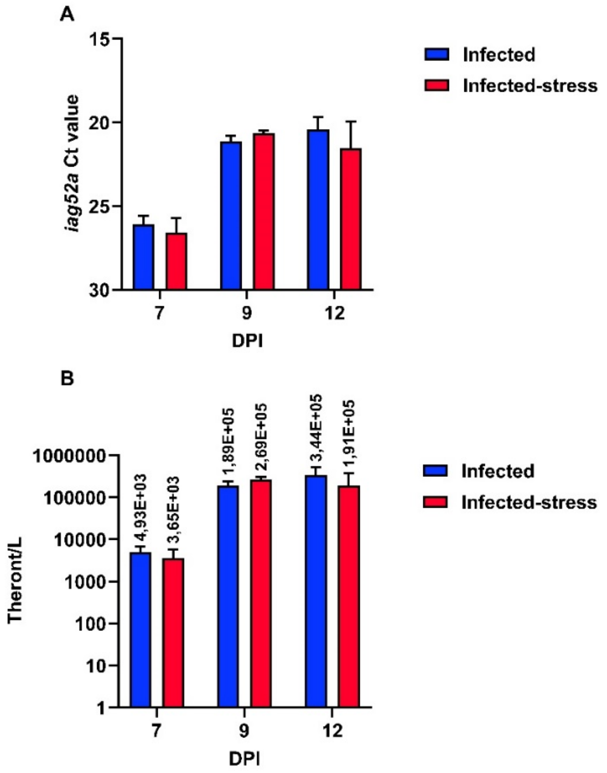

Figure S1. Monitoring of Ich *iag52a* water eDNA signal at 7, 9 and 12 dpi (A) and calculation of the theront concentration according to the Ich *iag52a* water eDNA signal (B).

Ich standard curve

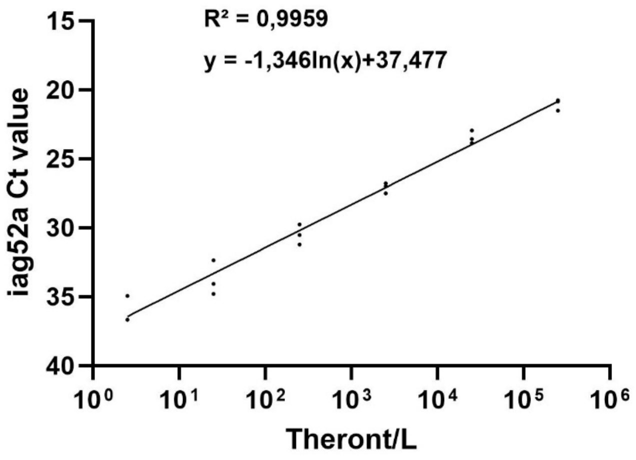

Figure S2. Standard curve of Ich *iag52a* water eDNA signal related with theront concentration.
